# A supergene in seaweed flies modulates male traits and female perception

**DOI:** 10.1098/rspb.2023.1494

**Published:** 2023-10-11

**Authors:** Swantje Enge, Claire Mérot, Raimondas Mozūraitis, Violeta Apšegaitė, Louis Bernatchez, Gerrit A. Martens, Sandra Radžiutė, Henrik Pavia, Emma L. Berdan

**Affiliations:** ^1^ Department of Marine Sciences, University of Gothenburg, Tjärnö, Sweden; ^2^ Département de biologie, Institut de Biologie Intégrative et des Systèmes (IBIS), Université Laval, Québec, Canada; ^3^ CNRS UMR 6553 Ecobio, Université de Rennes, OSUR, Rennes, France; ^4^ Department of Zoology, Stockholm University, Stockholm, Sweden; ^5^ Laboratory of Chemical and Behavioural Ecology, Institute of Ecology, Nature Research Centre, Vilnius, Lithuania; ^6^ Institute of Cell and Systems Biology of Animals, University of Hamburg, Hamburg, Germany

**Keywords:** supergene, disassortative mating, chemical communication, cuticular hydrocarbons, inversion, chemosensory genes

## Abstract

Supergenes, tightly linked sets of alleles, offer some of the most spectacular examples of polymorphism persisting under long-term balancing selection. However, we still do not understand their evolution and persistence, especially in the face of accumulation of deleterious elements. Here, we show that an overdominant supergene in seaweed flies, *Coelopa frigida*, modulates male traits, potentially facilitating disassortative mating and promoting intraspecific polymorphism. Across two continents, the *Cf-Inv(1)* supergene strongly affected the composition of male cuticular hydrocarbons (CHCs) but only weakly affected CHC composition in females. Using gas chromatography–electroantennographic detection, we show that females can sense male CHCs and that there may be differential perception between genotypes. Combining our phenotypic results with RNA-seq data, we show that candidate genes for CHC biosynthesis primarily show differential expression for *Cf-Inv(1)* in males but not females. Conversely, candidate genes for odorant detection were differentially expressed in both sexes but showed high levels of divergence between supergene haplotypes. We suggest that the reduced recombination between supergene haplotypes may have led to rapid divergence in mate preferences as well as increasing linkage between male traits, and overdominant loci. Together this probably helped to maintain the polymorphism despite deleterious effects in homozygotes.

## 1. Introduction

Complex multi-trait polymorphisms like colour morphs and specialized ecotypes are a fascinating aspect of intraspecific diversity. Such polymorphisms are increasingly known to be associated with supergenes, i.e. genomic regions harbouring linked combinations of alleles from recombination [1–3]. The loci in supergenes segregate together, acting as Mendelian loci and producing multi-trait phenotypes such as the mimetic morphs in the butterfly *Heliconius numata*, which differ in both coloration and patterning [4], reproductive morphs in ruff (*Philomachus pugnax*) which differ in plumage, size and behaviour [5,6] and flower distyly in multiple species of plants [7,8]. However, while the linked genetic architecture of supergenes, particularly when due to chromosomal inversions, helps to stabilize polymorphism it also can lead to increased accumulation of deleterious load [9–12] by reducing the efficacy of purifying selection [3,10,13]. Thus, the persistence of supergene polymorphism over long-time scales remains puzzling.

Understanding the maintenance of polymorphism is a key focus of supergene research and several different mechanisms of balancing selection have been identified in supergene systems [11,14]. One of these mechanisms, disassortative mating, appears to be quite prevalent [11]. Disassortative mating occurs when individuals preferentially mate with dissimilar phenotypes and is found in many classic supergene systems [13,15–18]. Disassortative mating is particularly adaptive in supergenes with deleterious mutations because those are generally private to one supergene arrangement, generating a heterozygote advantage [10,19–21]. Theory suggests that the evolution of such a mating system requires either (i) a self-referencing system where individuals use their own phenotype to choose a mate or (ii) tight linkage between mating signals and preferences [21,22]. By including many loci, supergenes are obviously good candidates for the latter situation. Yet, the underlying biological mechanisms and the genetic architecture of disassortative mating remain poorly known.

To better understand this form of mate choice and its role in supergene maintenance, we explored the traits and genetic basis of disassortative mating in relation to the *Cf-Inv(1)* supergene in the seaweed fly *Coelopa frigida*. *Cf-Inv(1)* is a large supergene spanning 10% of the genome [23] with two arrangements, termed *α* and *β*, resulting from three overlapping inversions [24]. The *Cf-Inv(1)* supergene affects multiple phenotypic traits such as development time, fitness on different substrates and adult size [25–27]. It is characterized by strong overdominance. In the wild, genotype frequencies deviate from Hardy–Weinberg and heterozygotes are found in excess (1.1–1.6× their expected frequency; [25,27,28]. In experimental populations, the egg-to-adult survival of homozygotes is reduced by 10–20% at low density and by 50–70% at high density when compared to heterozygote survival [29–31], suggesting that each supergene haplotype has accumulated a significant deleterious load. Non-random mating with respect to *Cf-Inv(1)* has been shown in several experimental and natural populations [25,29,32]. This may be explained in part by a higher success of large males, which are more likely to win competitions and successfully mount females without being dislodged [33]. While this process may underlie a proportion of disassortative mating (particularly between *ββ* females and large *αα* males), it does not seem to be the only process at play [25]. Several lines of evidence support pre- and post-copulatory female choice favouring reproduction with males exhibiting a different genotype at the supergene. First, disassortative mating was more frequent than assortative mating in semi-natural conditions for the three genotypes [34]. The same mating pattern was observed between opposite lines of supergene homozygotes with about 20–60% more disassortative choice than assortative choice, even when controlling for size [25]. Second, upon successive matings, which frequently happen in *C. frigida*, paternity was dominated by the sperm of the male with the opposite genotype [35]. Disassortative mate choice may have been selected because of the observed overdominance, and both mechanisms may contribute to the persistence of polymorphism.

The mechanisms underlying disassortative mate choice in *C. frigida* remain unknown. Males perform no courtship displays in this system: they mount females indiscriminately and are dislodged by the female via kicking, wing flicking or other manoeuvres in 30–50% of mating attempts [25,32,36]. Females receive no visual cues but the males rub the female's antennae with their forelegs during mounting [36] and females without antennae are less likely to mate while males with painted legs are more often dislodged [36]. Based on this, we hypothesized that chemical cues may play a role in sexual communication in this system. Cuticular hydrocarbons (CHCs) are a common mode of communication in insects and often facilitate mate choice [37,38]. Our previous work has demonstrated that CHC composition varies between sexes in *C. frigida*, making CHCs good candidate cues for sexual selection [39].

In this study, we investigated the hypothesis that chemical communication is conditioned by the supergene genotype *Cf-Inv(1)*, putatively explaining disassortative mating with respect to this overdominant supergene. To test this, we quantified differences in CHC profiles between *Cf-Inv(1)* genotypes in populations from two continents and predicted that male CHC profiles might show a stronger effect of supergene genotype than female profiles. We combined this with measurements of female perception of these compounds using gas chromatography–electroantennographic detection (GC–EAD). Finally, we sought to more thoroughly explore the genetic basis of male traits and female perception using differential expression analyses.

## 2. Methods

### (a) Collection and rearing of flies for CHC and EAD analysis

Wild *C. frigida* larvae were collected from Østhassel, Norway (58.07068, 6.64346) in March 2018 and transported to Tjärnö Marine Laboratory, Gothenburg University (Strömstad, Sweden) where they were allowed to mature on their natural wrack until all emerging adults were collected. Wild *C. frigida* adults were collected in September 2018 at Kamouraska, QC, Canada (47.56294, −69.87375) and transported to Laval University (Québec, Canada). Adults were allowed to lay eggs on standardized laboratory wrack (approx. 50% fucoids and 50% kelp) and this subsequent generation was used for CHC analysis (i.e. the focal generation). Laboratory conditions were standardized between Sweden and Canada and all flies were raised in a temperature-controlled room at 25°C with a 12 h/12 h light–dark cycle.

For the focal generations, as larvae pupated, they were transferred to individual 2 ml tubes with a small amount of cotton soaked in a solution of 0.5% mannitol. Two days after eclosure, flies were frozen at −80°C. To genotype each adult at the inversion, we extracted genomic DNA from one leg or the whole fly and performed a diagnostic SNP assay involving a PCR step amplifying the *Adh* gene, and a digestion step with two restriction enzymes targeting SNPs fixed between arrangements (described in [27]).

The focal generation of Norwegian flies for the EAD analysis was obtained from larvae collected in September 2021 from the same site (Østhassel, Norway) and genotyped by extracting DNA from one leg. The focal generation of Canadian flies for the EAD analysis was obtained from culture lines homozygous at the *Cf-Inv(1)* and descending from wild adults collected in July 2021 from the same site (Kamouraska, Québec). All flies were reared under the conditions described above. Adult flies were kept at 5°C in 2 ml tubes containing cotton soaked in a solution of 0.5% mannitol and a piece of seaweed for a maximum of two weeks before GC–EAD analysis.

### (b) CHC analysis

Frozen flies were allowed to defrost and dry for 10 min. Each fly was then placed in a 1.5 ml high recovery vial containing 300 µl of *n*-hexane, vortexed at a low speed for 5 s and extracted for 5 min. Afterwards, flies were removed from the vial and allowed to air dry before they were weighed. Extracts were evaporated until dryness under a stream of nitrogen and stored at −20° until GC–MS analysis. Before analysis, extracts were redissolved in 20 µl of *n*-hexane containing 1 µg ml^−1^ *n*-nonane (Sigma-Aldrich) as an internal standard and vortexed at maximum speed for 10 s. Extracts of about 25 flies per population (Norway and Canada), sex and genotype were analysed on a GC–MS (see electronic supplementary material for details).

The total ion chromatograms were quality checked before peak integration in OpenChrom and peak alignment using the R package ‘GCalignR’ [40]. The peak list was manually revised and filtered on minimum peak area and number of missing peaks before statistical analysis (see electronic supplementary material for details).

#### (i) Statistical analyses of CHC profiles

For the analysis of CHC profiles, peak areas were normalized on the internal standard peak area and the weight of the fly. For the multivariate analysis, all data were additionally mean centred and unit variance scaled. Extreme outliers were identified by deviations in the orthogonal and score distance in a PCA, and removed prior to analysis. Balanced sample sets were visualized with PCAs for Canadian females (*αα*/*αβ*/*ββ* = 22), Canadian males (*αα*/*αβ*/*ββ* = 22), Norwegian females (*αα*/*αβ*/*ββ* = 10) and Norwegian males (*αα*/*αβ*/*ββ* = 5). Differences between *αα* and *ββ* genotypes were assessed by OPLS-DA using the ‘ropls”-package in R [41] and PERMANOVA on Euclidean distances using the ‘vegan’ package in R [42]. OPLS-DA model significance was estimated by permutation (number of iterations = 999).

### (c) Gas chromatography and electroantennographic detection

We tested whether females could sense male CHCs using GC–EAD with Canadian male CHC extract for Canadian females and Norwegian male CHC extract for Norwegian females. The effluent from the column was split into two equal parts to simultaneously record the compounds by a flame ionization detector and the electrophysiologically response of fly antenna mounted to electroantennographic detector (EAD). We required five or more successful GC–EAD recordings to consider a compound electrophysiologically active. Full details of the GC–EAD procedure are found in the electronic supplementary material.

#### (i) Statistical analyses of perception

We analysed the GC–EAD data in three different ways. All approaches were done on Norwegian and Canadian flies separately as they were given extracts from their own populations. First, we searched for multivariate patterns in perception by performing a PCA on EAD signal intensities in µV. We supplemented this with two different univariate approaches. We used the DESeq2 1.26.0 framework to determine which compounds may have elicited different reactions from different genotypes [43]. We added 1 to every value in the matrix and used the likelihood ratio test to determine if a model with genotype performed better than the reduced model with only an intercept. We considered an adjusted *p*-value (FDR) < 10% to be significant. We also ran univariate tests using a GLM framework. As our data were overdispersed and contained many zeros, we opted for a zero-inflated negative binomial regression which performed better (lower dispersion) than a zero-inflated Poisson regression. We implemented this regression in R using the pscl package [44]. As with DESeq2, we used a likelihood ratio test to determine if our model was better than the null model and considered an adjusted *p*-value < 10% to be significant. For our final list of significant compounds, we used the overlap between the DESeq2 and GLM approaches.

#### (ii) Analysis of EAD active compounds

Retention indexes were calculated from the GC–EAD/FID chromatograms and translated into expected retention times in the GC–MS chromatograms of the CHC analysis. By comparison of the expected retention times together with the GC-FID elution patterns, the EAD active peaks were identified in the GC–MS readings and peak areas manually extracted. The peak areas were normalized on the internal standard peak area and the weight of the fly prior to analysis. For each EAD active compound, differences between genotypes within sex and differences between males and females were analysed using a GLM approach implemented in R with sex, genotype and their interaction as potential factors. Contrast statements to specifically test for the difference between *αα* and *ββ* in males and females were implemented *post hoc*.

### (d) Differential expression analysis

We used several methods to identify candidate genes for CHC biosynthesis (CHCB) and odorant detectors which are detailed in the electronic supplementary material. We used previously reported bulk RNA-seq data (see [45] for details), which contains whole animal RNA of *C. frigida* from several Swedish and Norwegian populations, to examine differential expression at candidate genes for CHCB as well as odorant detection (OD). We subset this dataset to only retain data from adults (three *αα* males, three *αα* females, five *ββ* males and six *ββ* females). While RNA was taken from non-virgin females, we expect the expression of OD to remain meaningful for mate choice because females mate several times in their lifetime [35]. We used previously generated count matrices and used DESeq2 [43] to determine differentially expressed genes between *αα* and *ββ* in males, females and a combined analysis with both sexes. After the DESeq2 analysis but before applying an FDR correction the results were subset to only include our candidate genes. Conventional thresholds (log_2_ fold change > 2, adjusted *p*-value (FDR) < 5%) were used to identify differentially expressed transcripts within our candidate subset.

#### (i) Analyses of clusters of OD transcripts

For examination of genomic locations, we subset our list to only include transcripts that had a blast match containing one of the following terms ‘odorant-binding protein’, ‘gustatory receptor’ or ‘odorant receptor’ or a pfam annotation of GOBP (PF01395). Proteins from the two pairs within *Cf-Inv(1)* (figure 3*b*) were predicted using transdecoder (http://transdecoder.github.io) implemented in Trinity [46] using the longest ORF option. Complete proteins were aligned using the EMBOSS Needle global aligner run on the EMBL-EBI website (https://www.ebi.ac.uk/Tools/psa/emboss_needle/) and visualized using Jalview [47]. Conservation between sequences was calculated using the AMAS method [48] and protein structure was predicted using Jpred4 (http://www.compbio.dundee.ac.uk/jpred/) [49].

Coverage, *F*_ST_ and *d*_xy_ between genotypes were extracted from previously published analyses [23], sub-setting the dataset to 144 *αα* and 144 *ββ* wild-caught *C. frigida.* Briefly, low-coverage whole-genome sequences were aligned to the reference genome (assembled from an *αα* individual), and coverage, polymorphism and differentiation were analysed in ANGSD.

## 3. Results and discussion

### (a) CHC composition differs between supergene genotypes in *C. frigida* males

To quantify the role of *Cf-Inv(1)* in CHC composition, we collected CHC profiles using gas chromatography and mass spectrometry (GC–MS) from a total of 279 *C. frigida* and analysed variation between sexes and genotypes*.* We included populations from both North America (Kamouraska, QC, Canada; 47.56294, −69.87375) and Europe (Østhassel, Norway; 58.07068, 6.64346). While the demographic and historic processes leading to the colonization of both coast of the Atlantic are yet unknown, the two geographical populations represent natural replicates of polymorphism persistence. The samples included 20–25 individuals of each sex, genotype (*αα*, *αβ*, *ββ*) and population combination although due to contamination, sample sizes for different analyses varied (see electronic supplementary material, table S1 for a full summary of sample sizes).

**Figure 1.**
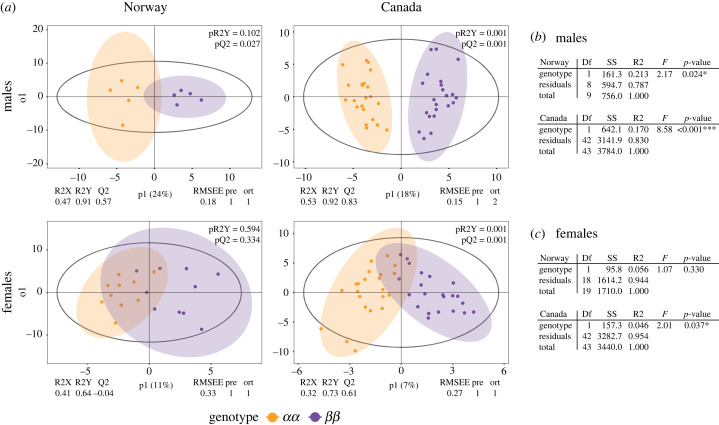
CHC composition varies by genotype in males but not females. (*a*) OPLS-DA analysis of CHC composition in Norwegian and Canadian populations. Figures are divided by population and sex and coloured by genotype: orange, *αα*; purple, *ββ*. (*b*) PERMANOVA results for males. (*c*) PERMANOVA results for females. Models were run separately for each population × sex combination. Only *αα* and *ββ* individuals were used in all analyses.

The overall CHC composition of males, visualized by PCA, showed some differentiation between the *αα* and *ββ* genotypes on both continents, with the *αβ* genotype as an intermediate phenotype (electronic supplementary material, figure S1). The effect of genotype (*αα* versus *ββ*) on CHC composition was further investigated by OPLS-DA and PERMANOVA (figure 1). OPLS-DA separates variation in CHC composition into variation correlated with genotype (the predictive component = between group variation) and other systemic variation uncorrelated with genotype (the orthogonal components = within group variation). In males, the two homozygous genotypes showed parallel separation by the OPLS-DA models on both continents (Norway R2Y = 0.91, Canada R2Y = 0.92; figure 1*a*), which also showed reliable predictive performances (Norway Q2 = 0.57, Canada Q2 = 0.83; figure 1*a*). Furthermore, 18% (Canadian males) and 24% (Norwegian males) of the total variation in CHC composition contributed to the separation of the *αα* and *ββ* genotypes. The effect of the supergene on CHC composition was further supported by the PERMANOVA results (figure 1*b*) demonstrating significant differences (*p* < 0.05) between *Cf-Inv(1)* genotypes in both Norwegian and Canadian males.

By contrast, very little variation in female CHC composition could be explained by *Cf-Inv(1)* genotype. The overall CHC composition of females, visualized by PCA, showed no differentiation between genotypes in either population (electronic supplementary material, figure S1). The OPLS-DA approach revealed that only 7% of the total variation in CHCs was associated with the genotype in Canadian females. Despite reliable predictive performance (Q2 = 0.61), the separation of the genotypes was less pronounced than in males (RY2 = 0.73; figure 1*a*). In the Norwegian females, no reliable predictive OPLS-DA model was obtained (RY2 = 0.64, Q2 =−0.04) demonstrating that genotype is a poor predictor for the variation in CHC composition. A PERMANOVA approach also demonstrated a significant difference (*p* < 0.05) between genotype in Canadian but not Norwegian females (figure 1*b*).

Overall, these results support the hypothesis that CHC composition between supergene genotypes varies much more strongly in males than in females. CHC profile thus appears to be a good candidate trait for female mate choice in this system, and is possibly the signal underlying disassortative mating in relation to *Cf-Inv(1)*. Intraspecific variation in pheromones is increasingly recognized in insects [50] and has been connected to supergenes or chromosomal rearrangements. For example, the *Sb* supergene in fire ants, which controls colony organization, also affects the CHC composition of queens [51–53]. Workers carrying the *Sb* supergene will accept queens or dummies with the correct CHC blend and reject queens without it [52,53]. Additionally, a segregating putative inversion in the European corn borer moth (*Ostrinia nubilalis*) contributes to intraspecific differentiation between insect pheromone strains [54]. By tightening linkage between multiple alleles, supergene architecture may be particularly favourable for maintaining intraspecific divergence in complex traits such as chemical signalling.

### (b) Male CHCs are perceived by *C. frigida* females, with slight differences between supergene genotypes

**Figure 2.**
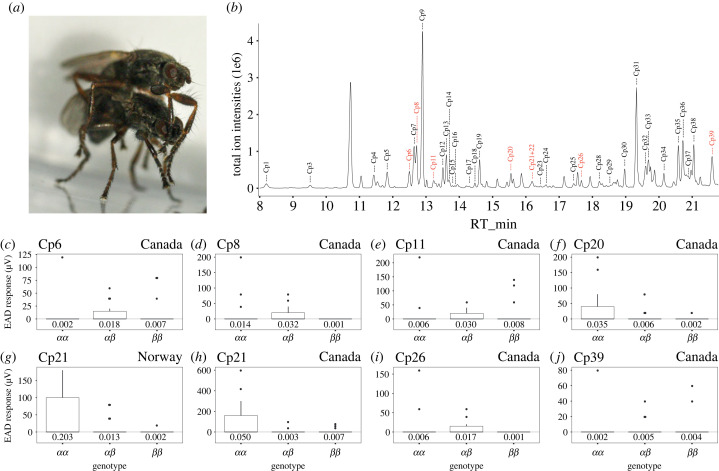
Female detection of CHCs varies by genotype. (*a*) Behaviour of *Coelopa frigida* during mating; note the legs of the male in direct contact with the female's antenna. Photo credit: Swantje Enge and Per Larsson. (*b*) Example chromatogram from an *αα* male with the identified EAD active peaks (Cp1–Cp39). Compounds showing significant differences in perception between genotypes are highlighted in red. (*c–j*) Boxplots of reaction (in μV) of female antennae to compounds with statistically significant differences between genotypes using both the DESeq2 and GLM approaches. Below each boxplot is the geometric mean for that group (all zeros have been changed to 0.001).

For CHCs to facilitate disassortative female choice, *C. frigida* females must be able to sense male CHCs, in particular the compounds that differ between genotypes*.* To investigate this, we measured female perception of male CHCs using GC–EAD. During mating, males place their forelegs over the female's head directly in contact with her antennae (figure 2*a*) so we focused on antennal perception. Reliable EAD readings could be obtained from 48 Norwegian females (13 *αα*, 20 *αβ* and 15 *ββ* genotypes) and 52 Canadian females (14 *αα*, 20 *αβ* and 18 *ββ* genotypes). In total, the females reacted to 39 compounds in the pooled male extracts (i.e. mixed extract from *αα*, *αβ* and *ββ* males) demonstrating that male CHCs are actively perceived by females and are relevant candidates for sexual communication. However, this data was highly variable, with individual females sensing between 1 and 15 compounds and most compounds being sensed by less than half of the females in each group (electronic supplementary material, figure S2).

To further understand sexual communication in relation to the supergene, we tested which cuticular compounds, among the ones perceived by females, showed differences in relative concentration between genotypes and sexes (table 1; electronic supplementary material, table S2). Of the 39 female EAD active compounds, 35 could be successfully identified in the recorded GC–MS chromatograms (figure 2*b*; electronic supplementary material, table S3). Three compounds had very low intensities in the fly extracts (Cp2 and Cp27) or uncertainties in the peak assignment (Cp10). Compounds 21 and 22 were further treated as one single compound as these peaks were not sufficiently resolved in the chromatograms. Eleven of the 35 identified candidate compounds showed a consistent, significant effect of sex between continents (table 1; electronic supplementary material, table S2). Moreover, several showed an effect of genotype or an interaction between genotype and sex (Norway—seven compounds; Canada—nine compounds; table 1). The effect of genotype was mostly restricted to males (table 1) concordant with our findings on the overall CHC composition. Restricting our analysis to males revealed that patterns between continents were somewhat consistent between populations. Six compounds showed significant and concordant effects in both populations, five compounds showed significant but opposing effects and an additional 16 were significant in only one population. Eight compounds showed no difference between males of different genotypes in either population. Intriguingly, all compounds showing increasing but opposing effects showed increased concentrations in *αα* males in Norway and *ββ* males in Canada. This matched the general patterns in both continents; 12/15 significant compounds in Norway were higher in *αα* males compared to 8/23 in Canada. The compounds displaying similar genotype/sex effects across continents may reflect either parallel evolution or that the initial coupling of female perception and male traits evolved before *C. frigida* spread to other continents while compounds differing between continents suggest that sexual selection for male CHC composition is likely also ongoing independently in both North America and Europe.

**Table 1.** Univariate analysis of 35 compounds quantified in adults and perceived by females. Values indicated under all are *t*-values from GLMs on log-transformed values of normalized peaks. Values under male and female are from *post hoc* contrasts done using the GLM results. Italics indicate a significant difference at *p* < 0.05. Corresponding adjusted *p*-values are found in electronic supplementary material, table S2. For sex, positive values indicate higher values in males compared to females and negative values indicate the opposite. For genotype positive values indicate higher values in *αα* individuals compared to *ββ* individuals and negative values indicate the opposite. For the genotype × sex interaction, a positive value indicates that *ββ* males had higher values than *αα* males and there was no significant genotype effect in females. A negative genotype × sex interaction indicates that *αα* males had higher values than *ββ* males and there was no significant genotype effect in females. The last column indicates compounds that are perceived differently between *αα* and *ββ* in at least one population, as assessed by a combination of methods.

	GC–MS: CHC quantified on adults body										GC–EAD: CHC perception by female antenna
	Norway					Canada					
	all			male	female	all			male	female	
CHC	sex	genotype	genotype × sex	genotype	genotype	sex	genotype	genotype × sex	genotype	genotype	genotype
Cp1	*5.46*	−0.24	−2.16	*3*.*29*	0.24	*5*.*45*	0.72	*−3*.*02*	*3*.*55*	−0.72	
Cp3	*3*.*02*	0.44	−1.43	1.44	−0.44	*5*.*58*	−0.52	−2.11	*3*.*53*	0.52	
Cp4	*2*.*78*	−1.49	−0.74	*2*.*54*	1.49	*3*.*79*	−1.11	−1.57	*3.33*	1.11	
Cp5	1.55	−0.55	0.21	0.25	0.55	0.63	−0.51	*3*.*46*	*−4*.*38*	0.51	
Cp6	2.09	0.05	−1.01	1.38	−0.05	*3*.*13*	−0.96	−1.36	*2*.*89*	0.96	* (Canada)
Cp7	0.96	−0.87	−0.95	2.21	0.87	*2*.*27*	−1.34	*−3*.*59*	*6*.*41*	1.34	* (Canada)
Cp8	0.15	−0.35	−0.36	0.86	0.35	1.32	−1.90	−1.44	*3*.*90*	1.90	
Cp9	−0.46	1.21	1.93	*−3*.*94*	−1.21	*−3*.*12*	−0.05	*5*.*39*	*−7*.*58*	0.05	
Cp11	−0.47	*−4*.*75*	−1.76	*7*.*23*	*4*.*75*	*−2*.*29*	0.58	1.24	*−2*.*34*	−0.58	* (Canada)
Cp12	0.06	0.33	−0.34	0.15	−0.33	−0.68	2.69	1.34	*−4*.*59*	−2.69	
Cp13	1.35	*−2*.*90*	−1.27	*4*.*69*	*2*.*90*	0.76	0.32	0.37	−0.84	−0.32	
Cp14	0.87	−0.72	−0.25	0.81	0.72	*2*.*66*	−1.47	−0.11	1.62	1.47	
Cp15	−0.92	*3*.*58*	−0.40	*−3*.*01*	*−3*.*58*	−0.97	3.12	−1.08	−1.56	−3.12	
Cp16	−2.14	0.57	0.67	−1.51	−0.57	*−3*.*88*	2.05	*5*.*00*	*−9*.*12*	−2.05	
Cp17	*−2*.*94*	*−3*.*16*	1.05	1.68	*3*.*16*	1.01	1.86	−0.34	−1.37	−1.86	
Cp18	−0.34	−0.34	−1.07	1.86	0.34	1.91	−0.89	−0.07	0.99	0.89	
Cp19	*−3*.*55*	−0.22	−0.54	0.98	0.22	*−4*.*63*	−0.27	1.09	−1.27	0.27	
Cp20	0.17	−1.86	−0.98	*2*.*51*	1.86	1.39	−1.83	0.62	0.95	1.83	* (Canada)
Cp21.22	2.16	−0.78	−1.64	*3*.*10*	0.78	1.91	−0.87	0.48	0.18	0.87	* (Canada/Norway)
Cp23	−1.88	−0.52	−1.05	2.01	0.52	−0.88	0.60	1.41	*−2*.*60*	−0.60	
Cp24	−1.97	−1.46	−0.57	2.27	1.46	*−5*.*50*	0.08	*2*.*62*	*−3*.*78*	−0.08	
Cp25	0.83	−1.19	−1.17	*2*.*87*	1.19	*2*.*32*	2.27	1.54	*−4*.*46*	−2.27	
Cp26	1.53	−1.75	−0.35	1.66	1.75	*3*.*73*	2.46	0.68	*−3*.*42*	−2.46	* (Canada)
Cp28	*4*.*98*	0.52	−2.61	*3.17*	−0.52	*2*.*95*	2.52	1.33	*−4*.*40*	−2.52	
Cp29	*−3*.*46*	−0.78	−0.85	1.98	0.78	−0.47	0.67	0.31	−1.10	−0.67	
Cp30	*2*.*61*	−0.29	−0.19	0.43	0.29	*3*.*59*	−0.35	*4*.*78*	*−6*.*40*	0.35	
Cp31	*9*.*97*	0.82	−2.32	*2*.*46*	−0.82	1.79	−0.23	*3*.*79*	*−5*.*14*	0.23	
Cp32	*8*.*92*	0.06	*−3*.*72*	*5*.*19*	−0.06	*5*.*59*	0.45	1.94	*−3*.*19*	−0.45	
Cp33	*3*.*96*	−0.19	−0.74	1.04	0.19	*9*.*06*	0.86	0.75	−1.92	−0.86	
Cp34	*5*.*27*	0.44	−1.25	1.22	−0.44	*7*.*93*	1.25	−0.87	−0.02	−1.25	
Cp35	*8*.*71*	−2.15	*−3*.*01*	*6*.*52*	2.15	*8*.*56*	−0.02	*−4*.*08*	*5*.*79*	0.02	
Cp36	*2*.*94*	0.83	−2.08	2.11	−0.83	2.13	−0.64	−0.98	2.02	0.64	
Cp37	−2.09	1.70	1.46	*−3*.*76*	−1.70	−1.67	0.85	1.30	*−2*.*70*	−0.85	
Cp38	*8*.*31*	1.22	*−3*.*97*	*4*.*40*	−1.22	*6*.*90*	−1.98	−0.27	*2*.*36*	1.98	
Cp39	*8*.*32*	0.78	−2.02	2.08	−0.78	1.60	−0.57	2.06	*−2*.*34*	0.57	* (Canada)

If CHC composition facilitates disassortative mating with respect to *Cf-Inv(1)*, we expect that chemical perception or signal processing in females may vary between genotypes. A PCA on female antenna responses (in µV) to all 39 compounds showed no group separation according to genotype in either continent (PCA, electronic supplementary material, figure S3). Although the number of compounds sensed by females did not vary between genotypes the coefficient of variation (CV = *σ*/*μ*) did. In both continents, the variance around the mean decreased with copies of *α* (i.e. CV *αα* > CV *αβ* > CV *ββ*; electronic supplementary material, figure S4). We supplemented these multivariate analyses with univariate analyses on all compounds that could be reliably identified and integrated in males and were sensed by the population being analysed (Canada: 24 compounds; Norway: 30 compounds). We also removed females that were outliers in the PCA analysis (Canada—four females; Norway—three females). The DESeq2 analysis on female antennal response revealed four compounds that were differently perceived between genotypes in Norwegian females and 17 compounds in Canadian females. The GLM approach on the same data identified one compound in Norwegian females and nine compounds in Canadian females. The overlap between the approaches yielded one compound (Cp21) that showed different patterns of chemoreception between the genotypes in both Norwegian and Canadian females and six additional compounds in the Canadian females (figure 2*c–j*). Five of these seven compounds differentially perceived by Canadian females differed in relative concentrations between Canadian *αα* and *ββ* males (table 1). The one compound differentially perceived by Norwegian females showed increased concentrations in Norwegian *αα* males when assessed in the poorly resolved peak together with Cp22.

To act as a mating signal, a compound has to be sensed, processed and ultimately result in a behavioural response [55]. Overall, our results show some potential differences in chemoreception between females but not overwhelming ones. This is partly due to the high inter-individual variation in the electric signal and the heterogeneous number of compounds perceived. Together these effects made it difficult to draw firm conclusions from our GC–EAD data, despite our large sample size. The observed variance may be both technical and biological. For example, if the distribution of olfactory sensilla is heterogeneous on the antenna, small differences placing the antenna towards odour stream eluting from GC could result in varying responses, as observed in *Dacus oleae* fly [56]. Thus, it is clear that examining chemoreception using multiple methodologies in combination with behavioural experiments will be critical to further assess the functional role of female-perceived CHCs in disassortative mating for this species.

### (c) Genes involved in biosynthesis and perception of CHCs are differentially expressed and genetically divergent between supergene genotypes

We sought to more thoroughly explore the basis of male traits and female chemoreception using differential expression analyses. Although the pathway of cuticular hydrocarbon biosynthesis (CHCB) is well established [38] a recent study revealed the effect of single genes on overall CHC composition is highly complex [57]. Thus, we took a more general approach; we identified 263 candidate transcripts for both CHCB and OD. This group included chemosensory receptors (gustatory and odorant), odorant-binding proteins (OBPs), sensory neuron membrane proteins, odorant degrading enzymes and ion channels, all noted as groups of interest in a recent review [58]. We tested the overall patterns of differential expression between genotypes in both sexes using previously published RNA-seq data from 17 European adults (3 *αα* ♀, 6 *ββ* ♀, 3 *αα* ♂, 5 *ββ* ♂) [45]. We performed three separate analyses: one with both sexes, one with males only and one with females only. Twenty-nine transcripts putatively acting on chemical communication were significantly differentially expressed between *αα* and *ββ*, with some overlap between analyses (electronic supplementary material, figure S5 and table S4). For the CHCB transcripts putatively involved in CHC synthesis, the signal was largely driven by males: differential expression between genotypes of 11/12 CHCB transcripts was restricted to males, and 7/12 CHCB transcripts were over-expressed in males compared to females (electronic supplementary material, table S4*a*). This is fully consistent with the observed larger effect on male CHC composition. Conversely, differential expression of 11/17 OD transcripts was significant in multiple analyses and not restricted to a single sex (electronic supplementary material, table S4*b*).

To ask whether the differentially expressed genes were located within *Cf-Inv(1)*, we mapped our candidate transcripts to v.1.0 of the *C. frigida* genome [23]. The two groups of genes showed strongly different patterns of localization. CHCB differentially expressed transcripts were widespread in the genome, with only one transcript mapping to the supergene, while nine OD transcripts mapped to *Cf-Inv(1).* This is significantly more than expected (52.9% of differentially expressed OD transcripts compared to 17% of all tested transcripts; Fisher's exact test *p* = 0.0053). Therefore, on the signal side, although the effect of *Cf-Inv(1)* on CHC composition was, as predicted, limited to males for both the phenotype and gene expression, it appears to be *trans* in relation to *Cf-Inv(1)* itself. This is in line with strong overall *trans* effects of *Cf-Inv(1)* in males particularly [45]. However, we still found clustering of CHCB loci, similar to what has been found in *Heliconius* butterflies [59]. As males and females share a genome, sex-specific changes in expression via cascading effects are more likely [60]. By contrast, on the reception side, the excess of OD genes within *Cf-Inv(1)* indicates a disproportionate effect of *Cf-Inv(1)* on chemoreception.

**Figure 3.**
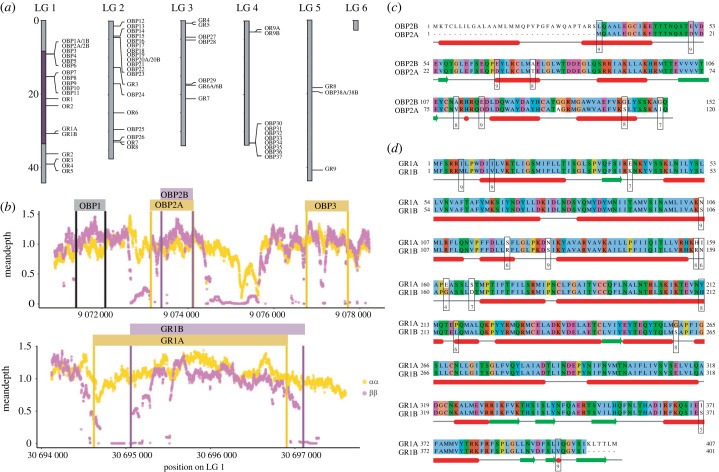
Divergence of odorant detection genes within the supergene. (*a*) Clusters of odorant detection genes identified in the *Coelopa frigida* genome. Putative genes were labelled as OR—odorant receptor, GR—gustatory receptor, OBP—odorant-binding protein. Numbers correspond to their order in the genome starting from LG1 position 1. Overlapping pairs were labelled with the same number and title but with A and B afterwards. The position of *Cf-Inv(1*) is shown in purple. Visualization was done with karyoploteR [64] (*b*) Coverage: average depth of sequencing across genotypes (yellow—*αα*, purple—*ββ*) at all positions along the focal genomic regions. Expected depth is around 1.1X. Bars indicate the position of the transcripts. (*c*,*d*) Protein alignment of OBP2A/B (*c*) and GR1A/B (*d*). Amino acids are coloured to show biochemical properties using the ClustalX colouring scheme. Protein structure predicted by Jpred is shown below with red bars indicating helices and green arrows indicating sheets. Below that is the AMAS conservation score with higher values indicating more conserved amino acids [48].

Next, we examined the distribution of OD genes across the genome regardless of expression status. We further subset our putative OD genes to look at odorant receptors (ORs), gustatory receptors (GRs) and OBPs as these form the base of chemoreception [61,62] and were found to be prime candidates for mate choice in *Heliconius* [63]. We found 63 transcripts that mapped to the genome (electronic supplementary material, table S5). The distribution of these transcripts was non-random, as several transcripts with similar or identical annotations were clustered at close proximity in blocks of 2–10 transcripts (figure 3*a*; electronic supplementary material, table S5). Such a clustering may be the result of tandem duplications and is frequently observed for OBPs and chemosensory receptors in insects [65–68]. Evolution by tandem duplication and subsequent divergence (e.g. the birth–death model of multi-gene families [69] is proposed to have generated the large and diverse OD gene families identified in insects [65,67,70,71]. While there was not an excess of OD genes within *Cf-Inv(1)* compared to its size, it had an excess of paired transcripts with overlapping coordinates (labelled with A/B; figure 3*a*).

We found six pairs of transcripts with overlapping coordinates, three of which mapped within *Cf-Inv(1)*. This pattern could be due to isoforms, exon duplications, errors in transcriptome assembly, or divergence. Two of these pairs (9.07 Mb and 30.69 Mb) were of particular interest as they showed opposite expression patterns (electronic supplementary material, table S4*b*). One transcript of each pair was assembled from an *αα* individual and the other transcript from a *ββ* individual, indicating that they probably represent alternative alleles of the same ancestral genes. Reusing previously published whole-genome sequences [23], we observed that both genomic regions are characterized by high genetic differentiation between *α* and *β* (*F*_ST_ = 0.96–0.97 and *d*_xy_ = 0.04–0.07 compared to *F*_ST_ = 0.87 and *d*_xy_ = 0.02 in *Cf-Inv(1)* overall), and heterogeneous coverage in *ββ* (figure 3*b*), which may reflect either low mapping of those sequences on an *αα* reference genome, or *ββ*-restricted deletions. Comparing the protein sequences of these pairs showed striking divergence in both amino acid identity and biochemical properties (figure 3*c*,*d*). The third pair within *Cf-Inv(1)* was also somewhat divergent, but less so (electronic supplementary material, figure S6). This indicates that the overlapping pairs within *Cf-Inv(1*) are due to divergence between supergene haplotypes. We note that after merging these pairs there was still a marginally significant excess of differentially expressed OD transcripts within *Cf-Inv(1)* (Fisher's exact test *p* = 0.0996). Thus, we found divergence in both coding sequences and expression. In most insects, diversification of tandem duplicates takes place between species [65,67], however, in *C. frigida*, we propose that early divergence in chemical signalling may have taken place within species, between arrangements of *Cf-Inv(1)*, although data from sister species is necessary to better understand the evolutionary history of those genes. A critical next step will be to perform ligand binding assays on these two alleles to determine if they differentially bind any of our focal CHCs. Overall, the duplication and divergence of OD genes between the two arrangements of the supergene is comparable to patterns observed between sister species of insects [72]. This is in line with fast evolution of OBPs and chemosensory genes [67] and suggests that supergenes provide a genetic architecture favouring the rapid divergent evolution of mate choice including at the intraspecific level.

### (d) Chemical signalling is a putative modality underlying disassortative mating and the persistence of supergene polymorphism

Theory predicts that disassortative mating requires either a self-referencing system where individuals use their own phenotype to choose a mate or tight linkage between mating signals and preferences [21,22]. As CHC profile differ between sexes but female CHCs vary little by genotype it is unlikely that a self-referencing mechanism guides mate choice relative to *Cf-Inv(1)*. Our results indicate that *Cf-Inv(1)* affects male CHC composition and probably plays a role in female chemoreception. However, more work remains to be done to understand differences in signal reception and processing in females. At this juncture, we cannot definitively link our results to female choice but based on our data we hypothesize that disassortative mating in this system may function via tight linkage between male mating signals and female preferences. The evolution of such a system in *C. frigida* supports theoretical predictions as overdominance creates a selective advantage for disassortative mating. Disassortative mating, and a linked architecture between trait and preference, are thus under strong selection, as choosing a mate with an alternate genotype ensures higher fitness. *Cf-Inv(1)* is strongly overdominant. Heterozygotes enjoy about a 25–75% increase in fitness due to a life-history trade-off between homozygotes [29] as well as about a 10–70% increase in survival (depending on environmental conditions) likely caused by masking of deleterious recessive alleles [10,19,20,73]. A potential pleiotropic effect of *Cf-Inv(1)*, on male traits, female chemoreception and the genes underlying overdominance, would result in a feedback loop that should strengthen disassortative mating and stabilize the polymorphism as suggested by mate choice theory [21,22] and speciation theory [74,75]. Although we cannot definitively tie differential CHC composition between *Cf-Inv(1)* genotypes with mate choice behaviour, our data point towards this tantalizing hypothesis.

## 4. Conclusion

Overall, we show that CHCs are a strong candidate for facilitating disassortative mating in *C. frigida*; males show strong differences in chemical composition by genotype, females can sense these compounds, and there are pieces of phenotypic and genetic evidence indicating that female chemoreception may vary between genotypes. More broadly, our findings highlight the importance of genetic architecture for the evolution of intraspecific diversity and the persistence of supergene-associated polymorphism. At a coarse scale, the reduction in effective recombination that favours the supergene is also putatively responsible for the accumulation of deleterious mutations leading to heterozygote advantage. The resulting selective pressure then favours disassortative mating. Potential linkage between the genes underlying male signal and the genes underlying female perception would provide an ideal architecture for the persistence of disassortative mating itself. Moreover, at a finer scale, the duplication of OD genes within the supergene region possibly contributed to the rapid divergence of signal reception. In this system, disassortative mating coupled with other mechanisms of balancing selection, such as spatially varying selection, preserve the coexistence of supergene haplotype across long-time scales, further enhancing the accumulation of divergence.

## Data Availability

GC–MS and GC–EAD data as well as scripts for the univariate analysis of perception, univariate analysis of EAD active compounds, and genetic analyses are all available on the Harvard Dataverse (https://dataverse.harvard.edu/dataverse/cfrigida). Raw RNA-seq reads are available on the NCBI SRA (Bioproject PRJNA746238). Supplementary material is available online [76].

## References

[1] Thompson MJ, Jiggins CD. 2014 Supergenes and their role in evolution. Heredity **113**, 1-8. (10.1038/hdy.2014.20) 24642887 PMC4815649

[2] Charlesworth D. 2016 The status of supergenes in the 21st century: recombination suppression in B atesian mimicry and sex chromosomes and other complex adaptations. Evol. Appl. **9**, 74-90. (10.1111/eva.12291) 27087840 PMC4780387

[3] Brelsford A, Purcell J, Avril A, Van PT, Zhang J, Brütsch T, Sundström L, Helanterä H, Chapuisat M. 2020 An ancient and eroded social supergene is widespread across Formica ants. Curr. Biol. **30**, 304-311. (10.1016/j.cub.2019.11.032) 31902719

[4] Joron M et al. 2011 Chromosomal rearrangements maintain a polymorphic supergene controlling butterfly mimicry. Nature **477**, 203-206. (10.1038/nature10341) 21841803 PMC3717454

[5] Küpper C et al. 2016 A supergene determines highly divergent male reproductive morphs in the ruff. Nat. Genet. **48**, 79-83. (10.1038/ng.3443) 26569125 PMC5218575

[6] Lamichhaney S et al. 2016 Structural genomic changes underlie alternative reproductive strategies in the ruff (*Philomachus pugnax*). Nat. Genet. **48**, 84-88. (10.1038/ng.3430) 26569123

[7] Huu CN, Keller B, Conti E, Kappel C, Lenhard M. 2020 Supergene evolution via stepwise duplications and neofunctionalization of a floral-organ identity gene. Proc. Natl Acad. Sci. USA **117**, 23 148-23 157. (10.1073/pnas.2006296117) 32868445 PMC7502755

[8] Gutiérrez-Valencia J et al. 2022 Genomic analyses of the Linum distyly supergene reveal convergent evolution at the molecular level. Curr. Biol. **32**, 4360-4371. (10.1016/j.cub.2022.08.042) 36087578

[9] Stolle E, Pracana R, Howard P, Paris CI, Brown SJ, Castillo-Carrillo C, Rossiter SJ, Wurm Y. 2019 Degenerative expansion of a young supergene. Mol. Biol. Evol. **36**, 553-561. (10.1093/molbev/msy236) 30576522 PMC6389315

[10] Berdan EL, Blanckaert A, Butlin RK, Bank C. 2021 Deleterious mutation accumulation and the long-term fate of chromosomal inversions. PLoS Genet. **17**, e1009411. (10.1371/journal.pgen.1009411) 33661924 PMC7963061

[11] Gutiérrez-Valencia J, Hughes PW, Berdan EL, Slotte T. 2021 The genomic architecture and evolutionary fates of supergenes. Genome Biol. Evol. **13**, evab057. (10.1093/gbe/evab057) 33739390 PMC8160319

[12] Jay P, Chouteau M, Whibley A, Bastide H, Parrinello H, Llaurens V, Joron M. 2021 Mutation load at a mimicry supergene sheds new light on the evolution of inversion polymorphisms. Nat. Genet. **53**, 288-293. (10.1038/s41588-020-00771-1) 33495598

[13] Tuttle EM et al. 2016 Divergence and functional degradation of a sex chromosome-like supergene. Curr. Biol. **26**, 344-350. (10.1016/j.cub.2015.11.069) 26804558 PMC4747794

[14] Berdan EL, Flatt T, Kozak GM, Lotterhos KE, Wielstra B. 2022 Genomic architecture of supergenes: connecting form and function. Phil. Trans. R. Soc. B **377**, 20210192. (10.1098/rstb.2021.0192) 35694757 PMC9189501

[15] Huynh LY, Maney DL, Thomas JW. 2011 Chromosome-wide linkage disequilibrium caused by an inversion polymorphism in the white-throated sparrow (*Zonotrichia albicollis*). Heredity **106**, 537-546. (10.1038/hdy.2010.85) 20571514 PMC2950911

[16] Horton BM, Hudson WH, Ortlund EA, Shirk S, Thomas JW, Young ER, Zinzow-Kramer WM, Maney DL. 2014 Estrogen receptor *α* polymorphism in a species with alternative behavioral phenotypes. Proc. Natl Acad. Sci. USA **111**, 1443-1448. (10.1073/pnas.1317165111) 24474771 PMC3910653

[17] Chouteau M, Llaurens V, Piron-Prunier F, Joron M. 2017 Polymorphism at a mimicry supergene maintained by opposing frequency-dependent selection pressures. Proc. Natl Acad. Sci. USA **114**, 8325-8329. (10.1073/pnas.1702482114) 28673971 PMC5547605

[18] Jay P, Whibley A, Frézal L, de Cara MÁR, Nowell RW, Mallet J, Dasmahapatra KK, Joron M. 2018 Supergene evolution triggered by the introgression of a chromosomal inversion. Curr. Biol. **28**, 1839-1845. (10.1016/j.cub.2018.04.072) 29804810

[19] Ohta T. 1971 Associative overdominance caused by linked detrimental mutations. Genet. Res. **18**, 277-286. (10.1017/S0016672300012684) 5158298

[20] Faria R, Johannesson K, Butlin RK, Westram AM. 2019 Evolving inversions. Trends Ecol. Evol. **34**, 239-248. (10.1016/j.tree.2018.12.005) 30691998

[21] Maisonneuve L, Chouteau M, Joron M, Llaurens V. 2021 Evolution and genetic architecture of disassortative mating at a locus under heterozygote advantage. Evolution **75**, 149-165. (10.1111/evo.14129) 33210282

[22] Kopp M et al. 2018 Mechanisms of assortative mating in speciation with gene flow: connecting theory and empirical research. Am. Nat. **191**, 1-20. (10.1086/694889) 29244561

[23] Mérot C et al. 2021 Locally adaptive inversions modulate genetic variation at different geographic scales in a seaweed fly. Mol. Biol. Evol. **38**, 3953-3971. (10.1093/molbev/msab143) 33963409 PMC8382925

[24] Aziz JB. 1975 Investigations into chromosomes 1, 2 and 3 of *Coelopa frigida* (Fab.). PhD Thesis, Newcastle University.

[25] Butlin RK, Read IL, Day TH. 1982 The effects of a chromosomal inversion on adult size and male mating success in the seaweed fly, *Coelopa frigida*. Heredity **49**, 51-62. (10.1038/hdy.1982.64)

[26] Gilburn AS, Day TH. 1994 Sexual dimorphism, sexual selection and the *α β* chromosomal inversion polymorphism in the seaweed fly, *Coelopa frigida*. Proc. Biol. Sci. **257**, 303-309. (10.1098/rspb.1994.0130)

[27] Mérot C, Berdan EL, Babin C, Normandeau E, Wellenreuther M, Bernatchez L. 2018 Intercontinental karyotype - environment parallelism supports a role for a chromosomal inversion in local adaptation in a seaweed fly. Proc. R. Soc. B **285**, 20180519. (10.1098/rspb.2018.0519) PMC6030540 29925615

[28] Day TH, Dawe C, Dobson T, Hillier PC. 1983 A chromosomal inversion polymorphism in Scandinavian populations of the seaweed fly, *Coelopa frigida*. Hereditas **99**, 135-145. 6580286 10.1111/j.1601-5223.1983.tb00738.x

[29] Mérot C, Llaurens V, Normandeau E, Bernatchez L, Wellenreuther M. 2020 Balancing selection via life-history trade-offs maintains an inversion polymorphism in a seaweed fly. Nat. Commun. **11**, 1-11. (10.1038/s41467-020-14479-7) 32015341 PMC6997199

[30] Butlin RK, Collins PM, Day TH. 1984 The effect of larval density on an inversion polymorphism in the seaweed fly *Coelopa frigida*. Heredity **52**, 415-423. (10.1038/hdy.1984.49)

[31] Gilburn AS, Crean CS, Day TH. 1996 Sexual selection in natural populations of seaweed flies: variation in the offspring fitness of females carrying different inversion karyotypes. Proc. R. Soc. B **263**, 249-256. (10.1098/rspb.1996.0039)

[32] Blyth JE, Gilburn AS. 2005 The effect of an inversion system and the time interval between matings on postcopulatory sexual selection in the seaweed fly, *Coelopa frigida*. Heredity **95**, 174-178. (10.1038/sj.hdy.6800713) 15999137

[33] Crean CS, Dunn DW, Day TH, Gilburn AS. 2000 Female mate choice for large males in several species of seaweed fly (Diptera: Coelopidae). Anim. Behav. **59**, 121-126. (10.1006/anbe.1999.1268) 10640374

[34] Day TH, Butlin RK. 1987 Nonrandom mating in natural poulations of the seaweed fly C*oelopa frigida*. Heredity **58**, 213-220. (10.1038/hdy.1987.35)

[35] Blyth JE, Gilburn AS. 2006 Extreme promiscuity in a mating system dominated by sexual conflict. J. Insect Behav. **19**, 447-455. (10.1007/s10905-006-9034-3)

[36] Day TH, Foster SP, Engelhard G. 1990 Mating behavior in seaweed flies (*Coelopa frigida*). J. Insect Behav. **3**, 105-120. (10.1007/BF01049198)

[37] Howard RW, Blomquist GJ. 2005 Ecological, behavioral, and biochemical aspects of insect hydrocarbons. Annu. Rev. Entomol. **50**, 371-393. (10.1146/annurev.ento.50.071803.130359) 15355247

[38] Blomquist GJ, Bagnères AG. 2010 Insect hydrocarbons: biology, biochemistry, and chemical ecology. Cambridge, UK: Cambridge University Press.

[39] Berdan EL, Enge S, Nylund GM, Wellenreuther M, Martens GA, Pavia H. 2019 Genetic divergence and phenotypic plasticity contribute to variation in cuticular hydrocarbons in the seaweed fly *Coelopa frigida*. Ecol. Evol. **9**, 12 156-12 170. (10.1002/ece3.5690) PMC6854331 31832150

[40] Ottensmann M, Stoffel MA, Nichols HJ, Hoffman JI. 2018 GCalignR: an R package for aligning gas-chromatography data for ecological and evolutionary studies. PLoS ONE **13**, e0198311. (10.1371/journal.pone.0198311) 29879149 PMC5991698

[41] Thevenot EA, Roux A, Xu Y, Ezan E, Junot C. 2015 Analysis of the human adult urinary metabolome variations with age, body mass index, and gender by implementing a comprehensive workflow for univariate and OPLS statistical analyses. J. Proteome Res. **14**, 3322-3335. (10.1021/acs.jproteome.5b00354) 26088811

[42] Dixon P. 2003 VEGAN, a package of R functions for community ecology. J. Veg. Sci. J. **14**, 927-930. (10.1111/j.1654-1103.2003.tb02228.x)

[43] Love MI, Huber W, Anders S. 2014 Moderated estimation of fold change and dispersion for RNA-seq data with DESeq2. Genome Biol. **15**, 550. (10.1186/s13059-014-0550-8) 25516281 PMC4302049

[44] Zeileis A, Kleiber C, Jackman S. 2008 Regression models for count data in R. J. Stat. Softw. **27**, 1-25.

[45] Berdan EL, Mérot C, Pavia H, Johannesson K, Wellenreuther M, Butlin RK. 2021 A large chromosomal inversion shapes gene expression in seaweed flies (*Coelopa frigida*). Evol. Lett. **5**, 607-624. (10.1002/evl3.260) 34917400 PMC8645196

[46] Haas BJ et al. 2013 De novo transcript sequence reconstruction from RNA-seq using the Trinity platform for reference generation and analysis. Nat. Protoc. **8**, 1494-1512. (10.1038/nprot.2013.084) 23845962 PMC3875132

[47] Waterhouse AM, Procter JB, Martin DM, Clamp M, Barton GJ. 2009 Jalview version 2- a multiple sequence alignment editor and analysis workbench. Bioinformatics **25**, 1189-1191. (10.1093/bioinformatics/btp033) 19151095 PMC2672624

[48] Livingstone CD, Barton GJ. 1993 Protein sequence alignments: a strategy for the hierarchical analysis of residue conservation. Bioinformatics **9**, 745-756. (10.1093/bioinformatics/9.6.745) 8143162

[49] Drozdetskiy A, Cole C, Procter J, Barton GJ. 2015 JPred4: a protein secondary structure prediction server. Nucleic Acids Res. **43**, W389-W394. (10.1093/nar/gkv332) 25883141 PMC4489285

[50] De Pasqual C, Groot AT, Mappes J, Burdfield-Steel E. 2021 Evolutionary importance of intraspecific variation in sex pheromones. Trends Ecol. Evol. **36**, 848-859. (10.1016/j.tree.2021.05.005) 34167852

[51] Eliyahu D, Ross KG, Haight KL, Keller L, Liebig J. 2011 Venom alkaloid and cuticular hydrocarbon profiles are associated with social organization, queen fertility status, and queen genotype in the fire ant *Solenopsis invicta*. J. Chem. Ecol. **37**, 1242-1254. (10.1007/s10886-011-0037-y) 22095515 PMC3800153

[52] Trible W, Ross K. 2016 Chemical communication of queen supergene status in an ant. J. Evol. Biol. **29**, 502-513. (10.1111/jeb.12799) 26644320

[53] Zeng H, Millar JG, Chen L, Keller L, Ross KG. 2022 Characterization of queen supergene pheromone in the red imported fire ant using worker discrimination assays. J. Chem. Ecol. **48**, 109-120. (10.1007/s10886-021-01336-0) 34850312

[54] Kozak GM, Wadsworth CB, Kahne SC, Bogdanowicz SM, Harrison RG, Coates BS, Dopman EB. 2017 A combination of sexual and ecological divergence contributes to rearrangement spread during initial stages of speciation. Mol. Ecol. **26**, 2331-2347. (10.1111/mec.14036) 28141898

[55] Yan H, Liebig J. 2021 Genetic basis of chemical communication in eusocial insects. Genes Dev. **35**, 470-482. (10.1101/gad.346965.120) 33861721 PMC8015721

[56] Crnjar R, Scalera G, Liscia A, Angioy A, Bigiani A, Pietra P, Barbarossa IT. 1989 Morphology and EAG mapping of the antennal olfactory receptors in *Dacus oleae*. Entomol. Exp. Appl. **51**, 77-85. (10.1111/j.1570-7458.1989.tb01216.x)

[57] Dembeck LM, Böröczky K, Huang W, Schal C, Anholt RR, Mackay TF. 2015 Genetic architecture of natural variation in cuticular hydrocarbon composition in *Drosophila melanogaster*. Elife **4**, e09861. (10.7554/eLife.09861) 26568309 PMC4749392

[58] Kohlmeier P, Billeter JC. 2023 Genetic mechanisms modulating behaviour through plastic chemosensory responses in insects. Mol. Ecol. **32**, 45-60. (10.1111/mec.16739) 36239485 PMC10092625

[59] Byers KJ et al. 2021 Clustering of loci controlling species differences in male chemical bouquets of sympatric Heliconius butterflies. Ecol. Evol. **11**, 89-107. (10.1002/ece3.6947) 33437416 PMC7790645

[60] Xu J et al. 2020 Regulation of olfactory-based sex behaviors in the silkworm by genes in the sex-determination cascade. PLoS Genet. **16**, e1008622. (10.1371/journal.pgen.1008622) 32520935 PMC7307793

[61] Fleischer J, Krieger J. 2018 Insect pheromone receptors–key elements in sensing intraspecific chemical signals. Front. Cell. Neurosci. **12**, 42563. (10.3389/fncel.2018.00425) PMC6255830 30515079

[62] Robertson HM. 2019 Molecular evolution of the major arthropod chemoreceptor gene families. Annu. Rev. Entomol. **64**, 227-242. (10.1146/annurev-ento-020117-043322) 30312552

[63] van Schooten B, Meléndez-Rosa J, Van Belleghem SM, Jiggins CD, Tan JD, McMillan WO, Papa R. 2020 Divergence of chemosensing during the early stages of speciation. Proc. Natl Acad. Sci. USA **117**:16 438-16 447. (10.1073/pnas.1921318117) PMC7371972 32601213

[64] Gel B, Serra E. 2017 karyoploteR: an R/bioconductor package to plot customizable genomes displaying arbitrary data. Bioinformatics **33**, 3088-3090. (10.1093/bioinformatics/btx346) 28575171 PMC5870550

[65] Vieira FG, Sánchez-Gracia A, Rozas J. 2007 Comparative genomic analysis of the odorant-binding protein family in 12 *Drosophila* genomes: purifying selection and birth-and-death evolution. Genome Biol. **8**, 1-16. (10.1186/gb-2007-8-11-r235) PMC2258175 18039354

[66] Gong DP, Zhang HJ, Zhao P, Xia QY, Xiang ZH. 2009 The odorant binding protein gene family from the genome of silkworm, *Bombyx mori*. BMC Genom. **10**, 1-14. (10.1186/1471-2164-10-1) PMC2722677 19624863

[67] Vieira FG, Rozas J. 2011 Comparative genomics of the odorant-binding and chemosensory protein gene families across the Arthropoda: origin and evolutionary history of the chemosensory system. Genome Biol. Evol. **3**, 476-490. (10.1093/gbe/evr033) 21527792 PMC3134979

[68] Dasmahapatra KK et al. 2012 Butterfly genome reveals promiscuous exchange of mimicry adaptations among species. Nature **487**, 94-98. (10.1038/nature11041) 22722851 PMC3398145

[69] Nei M, Rooney AP. 2005 Concerted and birth-and-death evolution of multigene families. Annu. Rev. Genet. **39**, 121-152. (10.1146/annurev.genet.39.073003.112240) 16285855 PMC1464479

[70] Sánchez-Gracia A, Vieira F, Rozas J. 2009 Molecular evolution of the major chemosensory gene families in insects. Heredity **103**, 208-216. (10.1038/hdy.2009.55) 19436326

[71] Duvaux L, Geissmann Q, Gharbi K, Zhou JJ, Ferrari J, Smadja CM, Butlin RK. 2015 Dynamics of copy number variation in host races of the pea aphid. Mol. Biol. Evol. **32**, 63-80. (10.1093/molbev/msu266) 25234705 PMC4271520

[72] Sánchez-Gracia A, Rozas J. 2008 Divergent evolution and molecular adaptation in the *Drosophila* odorant-binding protein family: inferences from sequence variation at the OS-E and OS-F genes. BMC Evol. Biol. **8**, 1-16. (10.1186/1471-2148-8-323) 19038039 PMC2631505

[73] Butlin RK, Day TH. 1985 Genic and karyotypic selection on an inversion polymorphism in the seaweed fly, *Coelopa frigida*. Heredity **54**, 267-274. (10.1038/hdy.1985.36)

[74] Servedio M. 2009 The role of linkage disequilibrium in the evolution of premating isolation. Heredity **102**, 51-56. (10.1038/hdy.2008.98) 18813328

[75] Butlin RK, Smadja CM. 2018 Coupling, reinforcement, and speciation. Am. Nat. **191**, 155-172. (10.1086/695136) 29351021

[76] Enge S, Mérot C, Mozūraitis R, Apšegaitė V, Bernatchez L, Martens GA, Radžiutė S, Pavia H, Berdan EL. 2023 A supergene in seaweed flies modulates male traits and female perception. Figshare. (10.6084/m9.figshare.c.6856554) PMC10565388 37817592

